# Ovine lameness in Ireland: a survey-based investigation of farmer reported prevalence, recognition, and treatment of lameness conditions

**DOI:** 10.1186/s13620-025-00313-3

**Published:** 2025-11-11

**Authors:** J. W. Delaney, E. T. Kelly, J. W. Angell, F. P. Campion

**Affiliations:** 1 Teagasc Animal and Grassland Research and Innovation Centre, Mellows Campus, Athenry, Co. , Galway, Ireland; 2 School of Veterinary Medicine, University College Dublin, Belfield, Ireland; 3 Department of Research and Innovation, Wern Vets Cyf, Unit 11, Lon Parcwr Industrial Estate, Ruthin, UK

**Keywords:** Sheep, Lameness, Survey, Contagious ovine digital dermatitis

## Abstract

**Background:**

Ovine lameness represents a significant production and welfare challenge to farmers and comprises two main categories, infectious and non-infectious lameness. The leading infectious lameness conditions are interdigital dermatitis (ID), footrot (FR) and contagious ovine digital dermatitis (CODD). The predominant types of non-infectious foot conditions include toe granulomas (TG) and shelly hoof (white line disease; SH).

There is a paucity of information available as to how Irish farmers treat infectious foot lesions in sheep. This study aimed to i) Establish farmer-reported prevalence of lameness in Irish sheep flocks, ii) Assess farmers’ ability to identify infectious and non-infectious lameness types, iii) Examine the treatment methods employed by farmers for managing and treating infectious lameness in sheep.

**Results:**

The survey was conducted opportunistically and non-randomly. Three hundred and sixty-three valid responses were gathered. The median overall farm lameness prevalence reported was 6.0% (IQR: 4.0%–10.0%). Respondents reported ID as the most common lameness condition with a median estimated prevalence of 10.0% (IQR: 4.0 – 15.0). Respondents identified 77.9%, 76.6%, 61.7%, 67.3% and 67.0% of ID, FR, CODD, TG and SH lesions correctly, respectively. Where the respective lesion was correctly identified 95.1%, 90.2%, 49.2%, 59.7% and 68.4% reported to have seen ID, FR, CODD, TG and SH lesions, respectively on their farm previously. Antibiotic aerosol was the most frequently utilised treatment for ID (71.3% [95% CI:65.2, 76.8%]) reported by survey respondents. Antibiotic injection was reported by respondents as the most used treatment for FR (72.2% [95% CI: 65.8,78.0%]) and CODD (85.1% [95% CI: 75.8, 91.8%]). Therapeutic foot trimming was practiced by 52.9% (95% CI:46.1,59.6%) of respondents for the treatment of FR.

**Conclusions:**

Interdigital dermatitis (ID) posed the greatest burden to Irish farmers, with a median farm prevalence of 10.0%, while footrot affected 90.2% of flocks (median 4.0%). Contagious ovine digital dermatitis, previously considered rare, was reported in 47% of flocks (median 3%). Farmers’ ability to correctly identify lesions varied from 79.9% for ID to 62.4% for CODD. Therapeutic foot-trimming, remain widely used within Irish flocks and was reported by 52.9% of respondents for the treatment of FR.

## Introduction/Background

Ovine lameness is both a production and welfare limiting condition [1] and is commonly cited by animal science specialists and farmers as one of the main concerns for both animal health and welfare [2, 3]. Lameness causing lesions can be categorised into two main categories, namely, infectious lameness and non-infectious lameness. Infectious lameness consists of three leading disease types: interdigital dermatitis (ID), footrot (FR) and contagious ovine digital dermatitis (CODD). The two most prominent non-infectious lameness conditions are shelly hoof (white line disease; SH) and toe granuloma’s (TG.) Collectively, ID and FR alone are estimated to cost the UK sheep industry £24 million annually due to the cost of treatment and preventative measures as well as their impact on production performance [4].

O’Brien, McHugh [5] reported lameness prevalences of 10.1% and 16.1% for ewes and lambs, respectively in a study encompassing data from 264 Irish flocks assessed between 2009 and 2015 by trained technicians. In the UK, Winter, Kaler [6] reported the geometric mean of ID, FR and CODD at 4.5%, 3.1% and 2.3%, respectively. In this UK study, ID and FR alone accounted for up to 68% of lameness cases [6]. Furthermore CODD, is now estimated to be present in 58% of UK flocks [7]. Granulomas were reported in 54–63% of flocks, with farmer-reported geometric mean lameness prevalence of in 4.1% in 2015. Shelly Hoof was reported in 58–76% of flocks, with geometric mean lameness prevalence of 3.3% in 2014 [8].

In past research, ID and FR were originally considered separate disease states with contrasting causative bacterial agents [9]. However, recent research carried out in the UK has found *Dichelobacter nodosus* to be the casual bacterial agent for both ID and FR [10]. The bacterium *F. necrophorum*, originally believed to be the initiating agent in both ID and FR infections, is now viewed as a secondary invader of the hoof post *D. nodosus* colonisation and can contribute to disease severity [11]. Furthermore, ID and FR increase the risk of sheep developing CODD; Staton, Angell [12] reported that 83.9% of CODD lesions arose from hooves initially infected with ID and/or FR. Although, the exact causal agent of CODD is still unknown, research indicates that its origins are likely poly-microbial [13]. *Treponema* spp., bacteria particularly, *T.medium*, *T.phagdenis* and *T.pedis* have been consistently associated with CODD lesions [14, 15] and appear a necessary cause of disease.

Although ID, FR and CODD have been linked, there is a difference in lesion presentation. Interdigital dermatitis presents as a ‘reddening’ and inflammation between the interdigital spaces, accompanied with a white exudate. FR is commonly associated with a foul-smelling odour and the necrosis of hoof tissue and capsule in the sole of the foot [16].

Contagious ovine digital dermatitis typically presents with a lesion on the coronary band of the hoof, which can be accompanied with hair loss along the coronary band region. The disease can progress to underrunning of the hoof wall in a vertical direction which can lead to the shedding of the hoof capsule [17]. In order to treat animals appropriately and reduce welfare and performance impacts, it is vital that farmers can recognise the type of lameness lesion they are treating, as different treatment methods are required for the successful resolution of the different diseases [18].

Foot bathing solutions and topical antibiotic sprays can be used for the treatment of ID [19], although the individual efficacy of singular use treatment products has not been specifically quantified in scientific literature. The dual use of an antibiotic injection and topical treatment is recommended in the treatment of FR [18]. Due to the low efficacy associated with the treatment of CODD with single use topical treatments, prompt treatment with a parental antibiotic is advised [20].

Within the Republic of Ireland there is a paucity of information on the prevalence of both infectious and non-infectious types of ovine lameness [7, 21]. While cases of CODD have been previously documented in Ireland, representing an emerging disease issue for Irish sheep flocks [22], previous research has not reported differences in flock-level prevalence or characterised the disease status of lameness cases reported [5]. The ability of Irish farmers to recognise infectious lameness is also currently un-reported. Consequently, disease prevalence levels and farmer treatment and control strategies are often estimated from UK studies, which may not reflect the Irish context [6, 23].

The objective of this study was multifaceted and had the following aims (i) establish farmer-reported prevalence of lameness in Irish sheep flocks, (ii) assess farmer ability to identify infectious and non-infectious lameness types, (iii) examine the treatment methods employed by farmers for managing and treating infectious lameness in sheep.

## Materials and methods

### Survey development and piloting

The survey was developed between November to December 2022 by E.K., J.A. and F.C., with J.D serving as the lead developer. The survey targeted Irish sheep farmers within the Republic of Ireland who own or are involved in the management of a sheep flock and consisted of 59 open, closed, and pictorial type questions. The survey was structured and designed in order to collect data relating to: i) farm size and farming system ii) description of respondents iii) ability of respondents to identify the five lameness conditions pictured, namely, ID, FR, CODD, SH and TG iv) respondents self-reported prevalence of lameness conditions on their farm v) methods employed by respondents for the treatment of lameness conditions on their farm, and vi) steps respondents implemented to prevent and control cases of ovine lameness on their farm.

The images of the lameness causing lesions were sourced from J.A., E.K., and J.D. There was a consensus among the authors that all images accurately depicted their respective lesions. When identifying lesions, respondents were presented with images of ID, FR, CODD, TG and SH followed by a brief written description. Questions were presented in multiple choice format and possible responses included: ID, FR, CODD, TG and SH, displayed in random order for each question. Respondents were also asked to estimate how many sheep per 100 sheep in their flock within the last year had presented with a lameness condition and how many sheep per 100 sheep in their flock within the last year presented with each individual lameness condition displayed for each photograph within the survey. Where a range in prevalence was provided by the respondent, the highest figure within that range was logged for analysis.

**Fig. 1:**
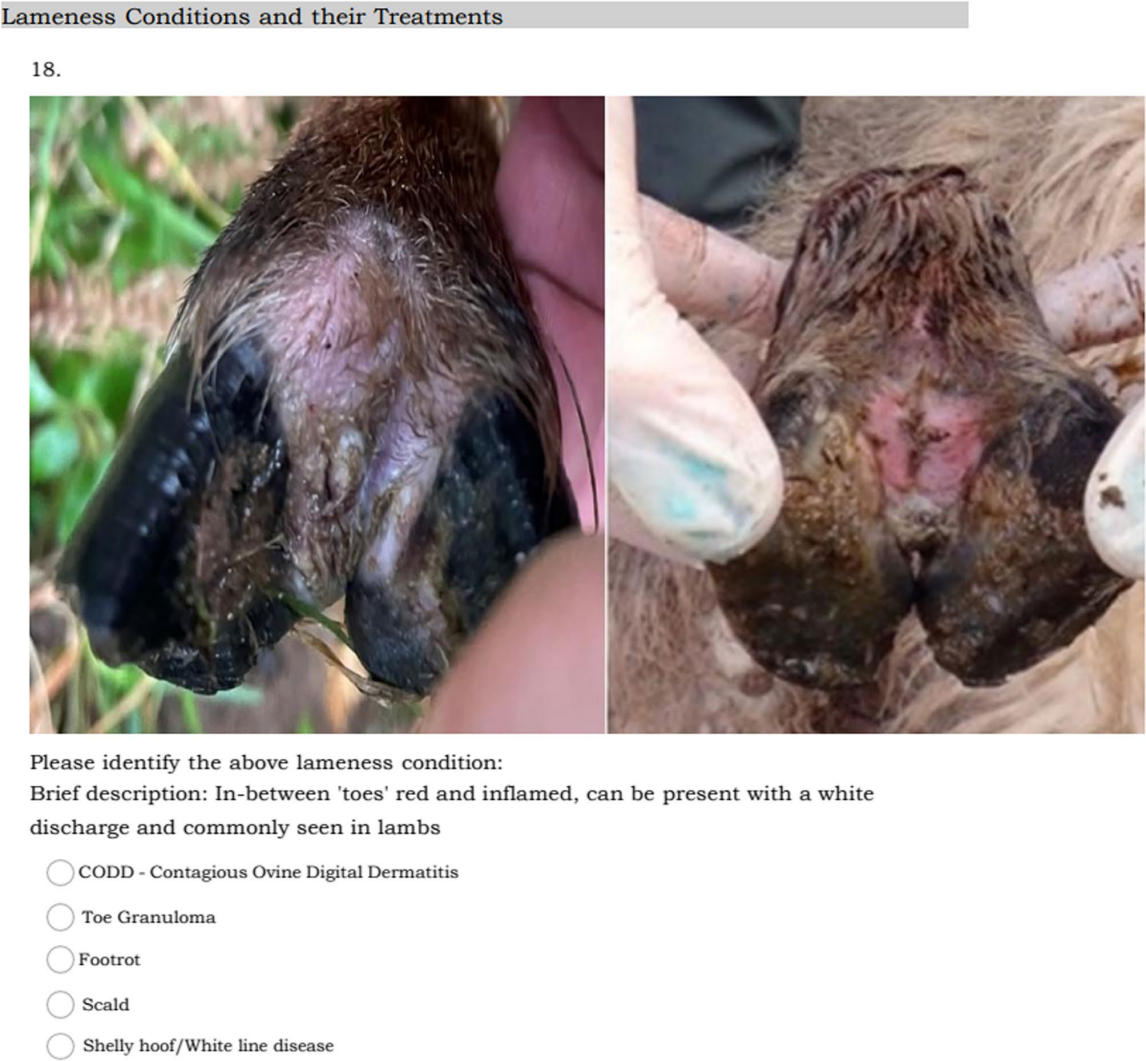
Example of question structure. The question displayed an ID lesion with multiple-choice format answers

An example of the survey structure is presented in Fig. 1. As respondents may recognise a lameness causing lesion but be unable to correctly name it, they were asked a follow-on question “have you seen this condition displayed on your farm before”. If respondents stated that they had seen the condition on their farm previously they progressed to the prevalence estimates and treatment types regardless of whether the lesion was correctly identified. However, if the respondents stated that they had never seen the lesion on farm before their subsequent answers for that lesion were excluded from analysis.

Due to the combination of treatments methods commonly used to treat cases of infectious lameness, multiple treatment options were permitted to be selected by respondents. Responses were only considered for analysis of treatment methods where the respondent correctly identified the lameness lesion.

The survey was initially pilot tested by eleven sheep researchers, specialists and technicians prior to the survey being made available. Post piloting Q12 was modified, and respondents were prompted to list the months in which their sheep were housed, if any. The survey was conducted opportunistically and non-randomly, with responses sought between January 2023 and May 2024. Time taken to complete the survey was estimated at approximately 10 minutes for respondents.

### Survey delivery –collection of responses

#### Online

Online responses were gathered via promotion of the survey through Teagasc (the agriculture and food development Authority; https://www.teagasc.ie/) social and National media and other National media outlets. Responses were collected on the online survey platform, ‘Survey monkey’ (www.surveymonkey.com). Responses gathered via ‘Survey Monkey’ were subsequently converted to Microsoft Excel 2016 [24].

#### In person

The survey was converted into a paper based format to allow for hybrid/mixed method delivery via both online and in-person routes of delivery. In-person responses were gathered through the convenience sampling of attendees at Teagasc sheep events located in Connaught (Donegal, Galway and Roscommon), Leinster (Carlow and Wexford), Ulster (Monaghan) and Munster (Tipperary). Reponses were also collected through attendance at sheep sales at five livestock marts, namely Carnaross (Co. Meath, Leinster) and Tullow (Co. Carlow, Leinster) Maam Cross (Co. Galway, Connacht), Raphoe (Co. Donegal, Ulster), and Kenmare (Co. Kerry, Munster). Responses gathered at in person events were collated via ‘Survey Monkey’ where all responses were subsequently converted into a Microsoft Excel 2016 [24].

### Data analysis of responses

Responses were not considered for analysis if questions relation to farm location and farm size were not answered or the location provided was located outside of the Republic of Ireland. Additionally, responses deemed invalid/unusable were also excluded from analysis. Due to the extensive number of options in a select number of multiple-choice type questions, response options for some survey questions, were merged, as per Table 1, to simplify interpretation and analysis. Following collation, SAS Version 9.4 was used for all data screening and subsequent analysis. All data were graphed and assessed for normality using the Shapiro-Wilk test. As data was non-normally distributed, right skewed, descriptive statistics were presented in median and inter quartile range to summarize central tendency. Proportions are presented with their corresponding 95% confidence intervals to describe the distribution of variables. The distribution of respondent reported lameness lesion prevalence was also graphed using SAS.

**Table 1:** Responses merged during data filtering for ease of analysis/interpretation

Survey Question	Original Response Options	Merged Response Options
Farm Enterprise	Sheep only	Sheep only (inc. Tillage/Forestry/Horses)
	Sheep/Suckler	Sheep/Suckler
	Sheep/Dry stock (Cattle)	Sheep/Other Cattle
	Sheep/Contact calf rearing	
	Sheep/Dairy	
	Sheep/Tillage	
	Other	
Ewe Breed	Belclare	Terminal
	Blue-faced Leicester	Maternal
	Charollais	Terminal/Maternal
	Cheviot	Terminal/Other
	Scottish Blackface	Maternal/Other
	Suffolk	Other
	Texel	
	Mule	
	Other	

## Results

### Respondent route and age

A total of 369 responses were collected. Of the 369 responses collected, 363 (98.4% [95%CI: 96.5, 99.4%]) were deemed to be usable following data screening. As per the exclusion parameters, one respondent did not list flock size, two respondents provided invalid responses, and three respondents did not list farm location or were based outside the Republic of Ireland. Not all respondents answered all questions contained within the survey. Due to the mixed method nature of the survey a response rate could not be calculated. Of the 363 usable responses, 248 (68.3% [95%CI:63.3, 73.1]) were collected through attendance at in-person events and 115 (31.7% [95%CI:26.9, 36.7]) were received via the online completion route. Respondents aged 30 years old or less had the highest representation in the respondent’s population accounting for 25.8% (n = 92, [95% CI: 21.1,30.3]) of respondents. Those aged between 31–45 years old comprised 23.5% of responses (n = 84, [95% CI: 19.1,28.0]). The total proportion of respondents aged 45 years old or under was 49.3% (95% CI: 44.0,54.6). Respondents aged between 46–55 years old accounted for accounted for 17.4% (n = 62, [95% CI: 13.4,21.3]), while those aged between 56–65 years old accounted for 21.0% (n = 75, [95% CI:16.8,25.3]) of responses. Respondents aged over 66 had the smallest representation of 12.3% (n = 44, [95% CI: 8.9,15.8]).

Response routes varied by respondent age. Respondents aged 30 years old or less, completed 52.2% of responses in person (95% CI: 41.6–62.6%) and 47.8% online (95% CI: 37.4–58.4%). In respondents aged 31–45 years, 63.1% responded in person (95% CI: 51.8–73.2%) compared with 36.9% online (95% CI: 26.8–48.2%). For those aged 46–55 years, 69.4% were in person (95% CI: 56.2–80.1%) and 30.6% online (95% CI: 19.9–43.8%). Among respondents aged 56–65 years, 80.0% completed the survey in person (95% CI: 68.9–88.0%) in contrast to 20.0% online (95% CI: 12.0–31.1%). For respondents aged over 66, 90.9% responded in person (95% CI: 77.4–97.0%) and 9.1% online (95% CI: 3.0–22.6%).

### Respondent time spent on farm

Part time farmers (i.e. those respondents who also had employment away from the farm) represented 58.3% (n = 211, [95% CI: 53.2,63.4]) of responses, while full time farmers accounted for of 41.7% (n= 151, 95% CI: 36.6, 46.8) of respondents.

### Location

**Fig. 2:**
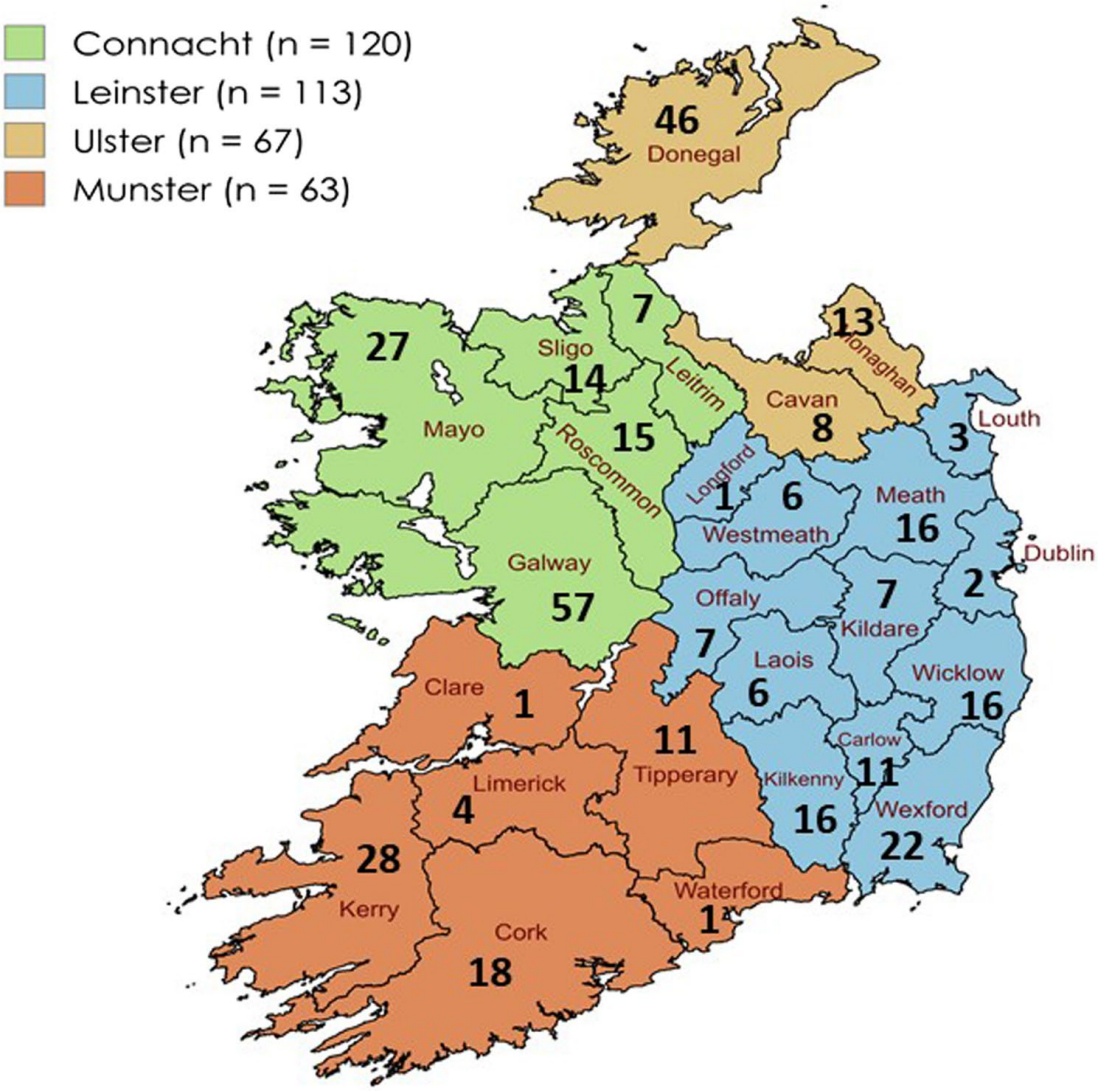
Distribution by county of number of survey respondents throughout the Republic of Ireland. Provinces represented by colour as per legend. Map created with MapChart.net and edited by authors

The highest proportion of respondents were from the province of Connaught, comprising of 33.1% of the total responses (n = 120, [95% CI: 28.2, 37.9]). Leinster accounted for 31.1% (n= 113, [95% CI: 26.3,35.9]). Connaught and Leinster combined account for 64.2% (n = 233, [95% CI: 59.2,69.1]) of responses. Ulster and Munster accounted for 18.5 (n =67, [95% CI: 14.4,22.5]) and 17.4% (n = 63, [95% CI:13.4,21.3]) of responses respectively. Respondents per county within the Republic of Ireland are displayed as per Fig. 2.

### Enterprise type

Farmers with sheep as their sole ruminant enterprise accounted for 42.7% of respondents (n = 155 [95% CI: 37.6,47.8]). Sheep/suckler beef cow enterprises represented 34.2% (n = 124, [95% CI: 29.3,39.1]) of responses, while enterprises consisting of sheep/other cattle constituted 23.1% (n = 84, [95% CI: 18.8,27.5]). Mixed sheep and cattle enterprises accounted for 57.3% (95% CI: 52.2,62.4) of the respondent population.

### Land type

Land type was defined into two types, Lowland or Hill/Upland. Three hundred and sixty-two respondents answered this question of which 68.5% (n = 248, [95% CI: 63.7,73.3]) and 31.5% (n =114, [95% CI: 26.7, 36.3]) identified as Lowland and Hill/Upland farmers respectively. The median farm size reported was 45 ha (IQR = 25.0, 74.0).

### Flock composition

The median flock size reported by survey respondents was 130 (IQR = 70.0, 220.0) ewes. Terminal breeds accounted for the highest frequency breed type (34.7% [95% CI: 29.8.39.7]) followed by crossbred terminal/maternal breeds (24.4% [95% CI: 19.9,28.8]) and hill breeds (19.6% [95% CI: 15.5,23.7]).

### Flock lameness prevalence

**Fig. 3:**
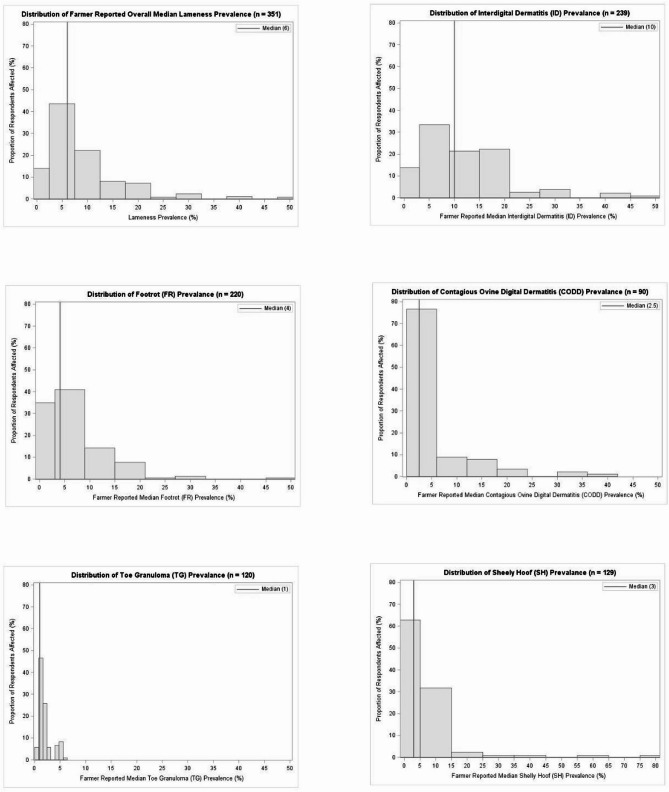
Comparison of distribution of respondent reported lameness prevalence. **a** Farm overall and (**b**) Interdigital Dermatitis (**c**) Footrot (**d**) Contagious Ovine Digital Dermatitis (**e**) Toe Granuloma and (**f**) Shelly Hoof. All respondents who provided overall farm average lameness prevalence are included in **a**. Only responses where lesions were correctly identified by respondents and an average prevalence number was reported are included in (**b**-**f**)

Respondents reported a median flock lameness prevalence of 6.0% (IQR: 4.0%–10.0%). The distribution of overall median farmer reported flock lameness prevalence is presented in Fig. 3(a).

### Lesion naming and prevalence

Respondents’ ability to correctly identify the five lameness conditions and the reported on-farm prevalence of each condition are shown as per Table 2. Also presented in Table 2 are the median prevalence of the associated lesions. Respondent’s ability to correctly identify the lameness conditions displayed ranged from 61.7% (95% CI: 56.3,67.2) for CODD to 77.9% (95% CI: 73.4,82.3) for ID. Where individual lesions were correctly identified by respondents, ID was found to be present on the highest proportion (95.1% [95% CI: 92.6,97.7]) of farms and had a median flock level prevalence of 10.0% (IQR: 4.0–17.0.0.0). In contrast, CODD was reported by respondents to be present on the lowest proportion of farms (49.2% [95% CI: 42.1,56.3]) and was reported to have a median prevalence of 2.5% (IQR: 1.0–5.0.0.0). The distribution of responses for the mean prevalence of the lameness conditions examined is illustrated in Fig. 3 (b-f).

**Table 2:** Respondent ability to identify lameness lesions and subsequent reported prevalence

Respondent Answer – number of respondents to correctly identify the condition (n) and corresponding respondent proportion % (95% CI)					
**Lesion Type **	Interdigital Dermatitis	Footrot	Contagious Ovine Digital Dermatitis	Toe Granuloma	Shelly Hoof
Interdigital Dermatitis	**268**	50	20	3	3
	77.9% *(73.5,82.3)*	14.5% (10.8,18.3)	5.8% (3.3,8.3)	0.9% (0.0,1.9)	0.9% (0.0,1.9)
Footrot	27	**258**	21	8	23
	8.0% (5.1,10.9)	**76.6% (72.0,81.1)**	6.2% (3.6,8.8)	2.4% (0.7,4.0)	6.8% (4.1,9.5)
Contagious Ovine Digital Dermatitis	19	42	**192**	21	37
	6.1% (3.4,8.7)	13.5% (9.7,17.3)	**61.7% (56.3,67.2)**	6.8% (3.9,9.6)	11.9% (8.3,15.5)
Toe Granuloma	8	35	20	**204**	36
	2.6% (0.8,4.5)	11.6% (7.9,15.2)	6.6% (3.8,9.4)	**67.3% (62.0,72.6)**	11.9% (8.2,15.5)
Shelly Hoof	5	54	15	24	**199**
	1.7% (0.2,3.2)	18.2% (13.8,22.6)	5.0% (2.5,7.6)	8.1% (5.0,11.2)	**67.0% (61.6,72.4)**
Percentage of overall farmers where lesion was reported					
Flocks where lesion 'seen on farm' (%)	90.2% (87.0,93.3)	83.1% (79.1,87.1)	47.5% (42.0,53.0)	60.2% (54.7,65.7)	65.3% (60.0,70.7)
Median farmer reported lesion prevalence (%)	10.0%** [4.0–15.0]**	4.0% [2.0–8.0]	3.0% [1.0–6.0]	2.0% [1.0–3.0]	3.0% [2.0–5.0]
Percentage of farmers where lesion was named correctly					
Flocks where lesion 'seen on farm' (%)	95.1% (92.6,97.7)	90.2% (86.6,93.9)	49.2% (42.1,56.3)	59.7% (52.6,66.5)	68.4% (61.9,74.9)
Median farmer reported lesion prevalence (%)	10.0% [4.0–17.0]	4.0% [2.0–8.0]	2.5% [1.0–5.0]	1.0% [1.0–2.0]	3.0% [2.0–5.0]

Proportion of respondents who could identify lesions displayed correctly. Percentage of overall farmers who reported lameness lesions present on farm and their subsequent prevalence without/with correct identification

*Values within () represent 95% confidence intervals

**Values within [] represent inter quartile range

### Treatment of infectious lameness

The treatment methods utilised by respondents to treat the infectious lameness conditions, where the lesion was correctly identified by the respondent are presented in Table 3. The use of an antibiotic aerosol was the most frequently utilised treatment for ID while the administration of an antibiotic injection was reported by respondents as the most used for the treatment of FR and CODD.

**Table 3:** Respondents for each treatment method used for the treatment of infectious lameness lesions

Treatment Method	Lesion Type		
	Interdigital Dermatitis	Footrot	Contagious Ovine Digital Dermatitis
	(n= 247)	(n = 223)	(n = 87)
Foot-trimming	26.7% (21.3,32.7%)	52.9% (46.1,59.6%)	36.8% (26.7,47.8%)
Aerosol	71.3% (65.2, 76.8%)	63.2% (56.5,69.6%)	50.6% (39.6,61.5%)
Footbath	66.4% (60.1,72.2%)	60.4% (53.6,66.8%)	32.2% (22.6,43.1%)
Antibiotic Injection	30.0% (24.3, 36.1%)	72.2% (65.8,78.0%)	85.1% (75.8,91.8%)

Corresponding 95% confidence intervals where the lesion was correctly identified (values in parentheses represent 95% confidence intervals)

## Discussion

This study is the first within the Republic of Ireland to investigate, via survey, the ability of farmers to identify ovine lameness causing lesions. It also examines the farmer reported prevalence of both infectious and non-infectious lameness causing lesions and assesses the treatment methods employed to treat cases of infectious lameness. Establishing this information is necessary for the Irish sheep industry. Firstly, it provides an estimate of the prevalence and types of lameness occurring within Irish sheep flocks. Secondly, it identifies gaps in the ability of farmers to recognise lameness lesions, aiding future knowledge transfer initiatives. Thirdly, it offers insight into the treatment practices currently employed by farmers. These findings can support future research in this area while also contributing to the development of more effective knowledge transfer strategies.

The median farm and flock size reported by survey respondents is in comparison to that of the national average as reported by the national farm survey [25]. The national average farm and flock size within the Republic of Ireland is 44.0 ha and 135 ewes, respectively [25]. Respondents to this survey reported a median farm size of 45.0 ha and a median flock size of 130 ewes.

In contrast to the national average of 50%, more respondents (58.1%) to this survey reported working in off farm employment (part-time farmers) [25]. Furthermore, 49.3% of respondents to this survey were aged 45 or under in comparison to 20.8% nationally [26], these younger, potentially more progressive farmers may be more actively engaged in knowledge transfer activities and also more familiar with the use of online technologies for data collection. This increased engagement could explain their higher representation amongst survey respondents, as survey responses were collected at knowledge transfer events as per the respondent sampling method. A greater representation of older farmers in the sample would likely have improved the representativeness of the survey population in comparison to national averages, and improved generalisability of the results.

There is limited data available regarding ovine lameness within the Republic of Ireland, with past studies such as O’Brien, McHugh [5] estimating lameness prevalence within experimental flocks. However, this study did not diagnose the causative conditions. The overall lameness prevalence reported by O’Brien, McHugh [5] within the experimental flocks when locomotion scored by a trained technician was 14.5%. This is in contrast to the overall farmer reported median farm lameness prevalence of 6.0% (IQR: 4.0%–10.0%) reported by respondents of this survey. Winter, Kaler [6] reported, that farmer estimation of farm lameness prevalence may be underreported when the overall flock lameness prevalence rises above 10%. Due to the varying methods in which previous authors calculated and reported overall farm lameness prevalence, a recent comparable overall farm lameness prevalence including both ewes and lambs is unavailable. Best, Roden [27] calculated a mean farmer reported lameness prevalence of 3.2% in ewes in the UK during a recent farmer survey. As this study is the first within the Republic of Ireland to report on the prevalence of individual lameness lesions within flocks and the ability of farmers to identify them, no direct comparisons can be made between Irish studies. Although, Lewis, Green [28] found comparable levels of lameness prevalence to this study in a study of 269 English flocks. The latter reported combined median lesion prevalence for ewes and lambs of 12%, 5%, 4%, 2% and 4% were reported for ID, FR, CODD, TG and SH, respectively.

Respondent ability to correctly identify lameness causing lesions in this study was comparable to findings by Kaler and Green [29], who reported 83% and 85% of respondents identified ID and FR correctly, respectively. In contrast a higher proportion of respondents to this survey were able to correctly identify CODD, TG and SH in comparison to the 36%, 43% and 28% rate reported by the respondents to Kaler and Green [29] for the aforementioned lesions, respectively. This may be due to the study by Kaler and Green [29] taking place over 15 years before that of the present study. During this time awareness as to lameness conditions and their identification has possibly improved particularly with more digital media available and knowledge transfer opportunities. Respondents to Kaler and Green [29], as in this study also most frequently misnamed ID, CODD and SH as FR. While FR and TG lesions were also most frequently misnamed as ID and SH in both studies, respectively.

In a notable result, both ID and FR were reported by farmers as present on greater than 90% of flocks. The proportion of flocks affected by ID and FR in this study is higher than that reported in a recent study by Lewis, Green [28] where 269 English farmers reported that ID and FR were present within 77% of flocks.

Despite being identified within Ireland [22] CODD was anecdotally thought not to be widespread across Irish flocks with limited cases of ovine lameness attributed to the disease. In contrast, survey respondents reported CODD present within 49.2% of flocks where correct identification was reported. Within the UK, CODD is now considered an endemic disease. Duncan, Angell [30] reported a between flock CODD prevalence of 67% for UK farmers.

There is a paucity of research as to the individual efficacy of foot bathing and topical antibiotic spray for the treatment of ID. Although with limited sample size, as acknowledged by the author, Jackson, Grove-White [31] detailed a 53% and 61% cure rate for ID at 42 days post treatment with five days of foot bathing in a 10% zinc sulphate solution for 15 min and five days of daily topical antibiotic spray, respectively. Grogono-Thomas, Wilsmore [32] reported a 77% efficacy rate for the treatment of FR whilst using a zinc sulphate footbath twice in total over a five-day period. Due to the topical nature of the footbath solution it is unable to penetrate deep into the hoof tissue where the causative pathogens of FR/CODD often establish [15]. Therefore, treatment with systemic parental antibiotics is recommended in order treat FR and CODD [20]. However, due to the superficial nature of ID, foot bathing has curative properties and has been found to be associated to lower the risk of increased flock level lameness when used to prevent ID [6, 33]. Foot-bathing, in past studies has been shown to have a limited efficacy in the treatment of CODD when used as a single treatment [13].

The results of this study show that foot-trimming is commonly used amongst survey respondents to treat ovine lameness. Foot-trimming was once a commonly used treatment and prevention measure for lameness [34]. However, as previous research has shown, the use of routine foot-trimming has been linked to the development of some non-infectious lameness conditions such as TG’s due to the damage caused to sensitive tissue beneath the hoof horn, which can occur during hoof trimming [8].

Trimming active lesions has been found to delay lesion healing [35] due to the aggravation of the lesion and potential to introduce secondary infection due to the removed protection provided by the hoof horn [36]. Foot-trimming can also contribute to the spread of infectious lameness conditions within the flock, as the causative bacteria of ID, FR and CODD have been isolated from hoof trimmers after the trimming of sheep affected by these conditions [37, 38]. Locher, Giger [37] detailed that where FR diseased sheep were trimmed, post trimming 89.5% of trimming knives tested positive for viable D. *nodosus.* Likewise, Sullivan, Blowey [38] identified CODD associated treponemes in 8 out of 9 trimming blades used to trim CODD diseased sheep. Therefore, the use of preventative whole flock foot-trimming or the trimming of lame sheep can lead to the spread of lameness causing bacteria

The limitations of this study are that the findings may not be fully representative of the national flock or allow for inferences of disease prevalence at national level. Due to the online component of respondent recruitment, the survey may have been skewed towards younger proportion of responses than that reflective of the national average. Additionally, due to the self-selecting sampling method of this survey, respondents who themselves are engaged with advisory or veterinary service in manging and control lameness issues may be more likely to have responded, biasing responses to more engaged or progressive farmers. An even geographical distribution of survey respondents was not achieved. However, counties with the highest sheep numbers were well represented in the survey. The four counties with the reported highest sheep numbers in Ireland are, Donegal, Mayo, Galway and Kerry [39] and they accounted for the top four survey location responses in this study with 43.5% of respondents. The higher number of flock keepers within these counties increased the likelihood of farmers responding to media promotion or attending in person events in these locations.

## Conclusion

This study is the first to report on the ability of Irish sheep farmers to identify lameness causing lesions, report flock level prevalence of both infectious and non-infectious lameness, and describe farmer reported treatment methods for infectious lameness. Interdigital dermatitis was found to pose the largest burden on farmers when managing and treating lameness with a median farm prevalence of 10.0% reported amongst survey respondents. Footrot was also reported as highly prevalent and was reported on 90.2% of flocks with a median prevalence of 4.0%. Contagious Ovine Digital Dermatitis, anecdotally believed to be found infrequently within a minority of Irish flocks is now prevalent and was reported in over 47% of respondent’s flocks with a median prevalence of 3%. Respondents were mixed in their ability to identify lameness lesions, with correct identifications ranging from 79.9% for ID to 62.4% for CODD. The results of this study indicate that the practice of therapeutic foot-trimming, a practice now linked to increased cure times, decreased cure rates, and the development of non-infectious lameness types continues on a large proportion of respondent’s flocks, with 52.9% of survey respondents reporting to foot-trimming cases of footrot as part of their lesion treatment methods. Further knowledge dissemination and training is required to educate farmers on best practice when treating and identifying lameness lesions.

## Data Availability

The datasets analysed during the current study are available from the corresponding author on reasonable request.

## References

[1] FAWC. Opinion on lameness in sheep. Farm Animal Welfare Council; 2011.

[2] Goddard P, Waterhouse T, Dwyer C, Stott A. The perception of the welfare of sheep in extensive systems. Small Ruminant Res. 2006;62(3):215–25.

[3] Marcone G, Carnovale F, Arney D, De Rosa G, Napolitano F. Relevance of animal-based indicators for the evaluation of sheep welfare as perceived by different stakeholders. Small Ruminant Res. 2022;217:106827.

[4] Nieuwhof GJ, Bishop S. Costs of the major endemic diseases of sheep in great Britain and the potential benefits of reduction in disease impact. Anim Sci. 2005;81(1):23–9.

[5] O’Brien A, McHugh N, Wall E, Pabiou T, McDermott K, Randles S, et al. Genetic parameters for lameness, mastitis and dagginess in a multi-breed sheep population. Animal. 2017;11(6):911–9. 27881209 10.1017/S1751731116002445

[6] Winter JR, Kaler J, Ferguson E, KilBride AL, Green LE. Changes in prevalence of, and risk factors for, lameness in random samples of english sheep flocks: 2004–2013. Prev Vet Med. 2015;122(1–2):121–8. 26435034 10.1016/j.prevetmed.2015.09.014

[7] Dickins A, Clark CCA, Kaler J, Ferguson E, O’Kane H, Green LE. Factors associated with the presence and prevalence of contagious ovine digital dermatitis: A 2013 study of 1136 random english sheep flocks. Prev Vet Med. 2016;130:86–93. 27435650 10.1016/j.prevetmed.2016.06.009

[8] Reeves MC, Prosser NS, Monaghan EM, Green LE. Footbathing, formalin and foot trimming: the 3Fs associated with granulomas and Shelly hoof in sheep. Vet J. 2019;250:28–35. 31383417 10.1016/j.tvjl.2019.06.002

[9] Winter A. Lameness in sheep 2. Treatment and control. Pract. 2004;26(3):130–9.

[10] Witcomb LA, Green LE, Kaler J, Ul-Hassan A, Calvo-Bado LA, Medley GF, et al. A longitudinal study of the role of Dichelobacter nodosus and Fusobacterium necrophorum load in initiation and severity of footrot in sheep. Prev Vet Med. 2014;115(1):48–55. 24703249 10.1016/j.prevetmed.2014.03.004 PMC4029074

[11] Clifton R, Giebel K, Liu NLBH, Purdy KJ, Green LE. Sites of persistence of Fusobacterium necrophorum and Dichelobacter nodosus: a paradigm shift in Understanding the epidemiology of footrot in sheep. Sci Rep. 2019;9(1):14429. 31594981 10.1038/s41598-019-50822-9 PMC6783547

[12] Staton GJ, Angell JW, Grove-White D, Clegg SR, Carter SD, Evans NJ, Duncan JS. Contagious ovine digital dermatitis: A novel bacterial etiology and lesion pathogenesis. Front Veterinary Sci. 2021;8:722461. 10.3389/fvets.2021.722461 PMC8496452 34631855

[13] Bernhard M, Frosth S, König U. First report on outbreaks of contagious ovine digital dermatitis in Sweden. Acta Vet Scand. 2021;63(1):29. 34399828 10.1186/s13028-021-00595-x PMC8369363

[14] Sullivan L, Clegg S, Angell J, Newbrook K, Blowey R, Carter S, et al. High-level association of bovine digital dermatitis Treponema spp. With contagious ovine digital dermatitis lesions and presence of Fusobacterium necrophorum and Dichelobacter nodosus. J Clin Microbiol. 2015;53(5):1628–38. 25740778 10.1128/JCM.00180-15 PMC4400778

[15] Tegtmeyer PC, Staton GJ, Evans NJ, Rohde J, Punsmann TM, Ganter M. First cases of contagious ovine digital dermatitis in Germany. Acta Vet Scand. 2020;62(1):46. 32854737 10.1186/s13028-020-00544-0 PMC7457254

[16] Winter A. Lameness in sheep 1. Diagnosis Pract. 2004;26(2):58–63.

[17] Angell J, Blundell R, Grove-White D, Duncan J. Clinical and radiographic features of contagious ovine digital dermatitis and a novel lesion grading system. Vet Rec. 2015;176(21):544. 25861825 10.1136/vr.102978

[18] Green L, Clifton R. Diagnosing and managing footrot in sheep: an update. Pract. 2018;40(1):17–26.

[19] Green L, Wassink G, Kaler J, Hawker E, Daniels S, Thomas RG. Practicalities of lameness management in sheep. UK Vet Livest. 2008;13(7):50–4.

[20] Duncan J, Angell J. Control of infectious lameness in sheep. Livestock. 2019;24(5):246–51.

[21] Angell J, Duncan J, Carter S, Grove-White D. Farmer reported prevalence and factors associated with contagious ovine digital dermatitis in wales: a questionnaire of 511 sheep farmers. Prev Vet Med. 2014;113(1):132–8. 24207114 10.1016/j.prevetmed.2013.09.014

[22] Sayers G, Marques PX, Evans NJ, O’Grady L, Doherty ML, Carter SD, Nally JE. Identification of spirochetes associated with contagious ovine digital dermatitis. J Clin Microbiol. 2009;47(4):1199–201. 19204100 10.1128/JCM.01934-08 PMC2668357

[23] Kaler J, Green L. Farmers’ practices and factors associated with the prevalence of all lameness and lameness attributed to interdigital dermatitis and footrot in sheep flocks in England in 2004. Prev Vet Med. 2009;92(1–2):52–9. 19735953 10.1016/j.prevetmed.2009.08.001

[24] Corporation M. Microsoft Excel 2016 ed. 2016.

[25] Dillion E, Donnellan T, Moran B, Lennon J. Teagasc National Farm Survey. 2023.

[26] (CSO) CSO. Census of Agriculture 2020 - Preliminary Results: Demographic profile of farm holders: CSO; 2020 [Cited 2024 Dec 20]Available from: https://www.cso.ie/en/releasesandpublications/ep/p-coa/censusofagriculture2020-preliminaryresults/demographicprofileoffarmholders.

[27] Best CM, Roden J, Pyatt AZ, Behnke M, Phillips K. Uptake of the lameness Five-Point plan and its association with farmer-reported lameness prevalence: A cross-sectional study of 532 UK sheep farmers. Prev Vet Med. 2020;181:105064. 32593081 10.1016/j.prevetmed.2020.105064

[28] Lewis KE, Green M, Clifton R, Monaghan E, Prosser N, Nabb E, Green L. Footbathing and foot Trimming, and no quarantine: risks for high prevalence of lameness in a random sample of 269 sheep flocks in England, 2022. Animals. 2024;14(14):2066. 39061528 10.3390/ani14142066 PMC11273439

[29] Kaler J, Green L. Naming and recognition of six foot lesions of sheep using written and pictorial information: a study of 809 english sheep farmers. Prev Vet Med. 2008;83(1):52–64. 17688961 10.1016/j.prevetmed.2007.06.003

[30] Duncan JS, Angell JW, Grove-White D, Walsh TR, Seechurn N, Carter S, Evans N. Impact of research on contagious ovine digital dermatitis on the knowledge and practices of UK sheep farmers and veterinarians. Vet Rec. 2022;190(1):no–no. 10.1002/vetr.674 34192349

[31] Jackson A, Grove-White DH, Angell JW, Duncan JS. Comparison of clinical cure rates from footrot and contagious ovine digital dermatitis using zinc sulphate foot bathing and topical oxytetracycline: A randomised trial. Vet Rec. 2023;193(6):e3116. 37308289 10.1002/vetr.3116

[32] Grogono-Thomas R, Wilsmore AJ, Simon AJ, Izzard KA. The use of long-acting Oxytetracycline for the treatment of ovine footrot. Br Vet J. 1994;150(6):561–8. 7850446 10.1016/S0007-1935(94)80041-3

[33] Wassink GJ, Green LE, Grogono-Thomas R, Moore LJ. Risk factors associated with the prevalence of interdigital dermatitis in sheep from 1999 to 2000. Vet Rec. 2004;154(18):551–5. 15143999 10.1136/vr.154.18.551

[34] Green L, Wassink G, Grogono-Thomas R, Moore L, Medley G. Looking after the individual to reduce disease in the flock: a binomial mixed effects model investigating the impact of individual sheep management of footrot and interdigital dermatitis in a prospective longitudinal study on one farm. Prev Vet Med. 2007;78(2):172–8. 17092589 10.1016/j.prevetmed.2006.09.005

[35] Kaler J, Daniels S, Wright J, Green L. Randomized clinical trial of long-acting oxytetracycline, foot trimming, and flunixine Meglumine on time to recovery in sheep with footrot. J Vet Intern Med. 2010;24(2):420–5. 20051002 10.1111/j.1939-1676.2009.0450.x

[36] Lovatt F, Page P. Sheep lameness: risks, causes, treatment and management. 2017.

[37] Locher I, Giger L, Frosth S, Kuhnert P, Steiner A. Potential transmission routes of Dichelobacter nodosus. Vet Microbiol. 2018;218:20–4. 29685216 10.1016/j.vetmic.2018.03.024

[38] Sullivan LE, Blowey RW, Carter SD, Duncan JS, Grove-White DH, Page P, et al. Presence of digital dermatitis Treponemes on cattle and sheep hoof trimming equipment. Vet Rec. 2014;175(8):201. 24821857 10.1136/vr.102269

[39] DAFM. Ireland National Sheep and Goat Census December 2023. 2024.

